# A Statistical Model for Estimating Maternal-Zygotic Interactions and Parent-of-Origin Effects of QTLs for Seed Development

**DOI:** 10.1371/journal.pone.0003131

**Published:** 2008-09-04

**Authors:** Yanchun Li, Cintia M. Coelho, Tian Liu, Song Wu, Jiasheng Wu, Yanru Zeng, Youchun Li, Brenda Hunter, Ricardo A. Dante, Brian A. Larkins, Rongling Wu

**Affiliations:** 1 School of Forestry and Biotechnology, Zhejiang Forestry University, Lin'an, Zhejiang, People's Republic of China; 2 Agricultural Ecology Research Institute, Fujian Academy of Agricultural Science, Fuzhou, Fujian, People's Republic of China; 3 Department of Plant Sciences, University of Arizona, Tucson, Arizona, Unites States of America; 4 Human Genetics Group, Genome institute of Singapore, Singapore, Singapore; 5 Department of Statistics, University of Florida, Gainesville, Florida, United States of America; 6 Department of Operations Research and Financial Engineering, Princeton University, Princeton, New Jersey, United States of America; University of Chicago, United States of America

## Abstract

Proper development of a seed requires coordinated exchanges of signals among the three components that develop side by side in the seed. One of these is the maternal integument that encloses the other two zygotic components, i.e., the diploid embryo and its nurturing annex, the triploid endosperm. Although the formation of the embryo and endosperm contains the contributions of both maternal and paternal parents, maternally and paternally derived alleles may be expressed differently, leading to a so-called parent-of-origin or imprinting effect. Currently, the nature of how genes from the maternal and zygotic genomes interact to affect seed development remains largely unknown. Here, we present a novel statistical model for estimating the main and interaction effects of quantitative trait loci (QTLs) that are derived from different genomes and further testing the imprinting effects of these QTLs on seed development. The experimental design used is based on reciprocal backcrosses toward both parents, so that the inheritance of parent-specific alleles could be traced. The computing model and algorithm were implemented with the maximum likelihood approach. The new strategy presented was applied to study the mode of inheritance for QTLs that control endoreduplication traits in maize endosperm. Monte Carlo simulation studies were performed to investigate the statistical properties of the new model with the data simulated under different imprinting degrees. The false positive rate of imprinting QTL discovery by the model was examined by analyzing the simulated data that contain no imprinting QTL. The reciprocal design and a series of analytical and testing strategies proposed provide a standard procedure for genomic mapping of QTLs involved in the genetic control of complex seed development traits in flowering plants.

## Introduction

In flowering plants, double fertilization of the female gametophyte by the two sperm cells of a pollen grain produces the diploid embryo and the triploid endosperm enclosed within the maternal tissue of the integuments. Thus, proper development of a seed depends on three different growth programs: those of the diploid maternal integument and the new-generation zygotic embryo and endosperm [1]. It has well been recognized that genes play a central role in directing each of these programs to determine the growth rate and final size of the seed [2]–[6]. A number of mutations were detected to be involved in integument development [7], [8], and genes affecting embryo and endosperm pattern formation have also been observed [9]–[11]. Some of these genes function by regulating the interactions and coordinations between different cell types within maternal-zygotic interfaces in the seed, but the relative contributions of the maternal and zygotic genomes and the nature of how these two types of genomes communicate to coordinate seed growth are poorly understood.

Genomic imprinting has been thought to play a role in regulating the interactions between maternal and zygotic tissues in the seed. Genomic imprinting is the process responsible for the generation of functional differences between maternally- and paternally-derived alleles at the same gene [12]–[15]. In flowering plants, many studies by inter-ploidy crosses and other experimental approaches showed parental origin-dependent differences of genes located on homologous chromosomes during seed development [16]. MEDEA [MEA] was the first gene in plants where expression was observed to depend on the parental origin of the allele; only maternal MEA alleles operate at the MEA locus during early seed development [17]. Since then, an increasing number of imprinted genes have been identified in mediating seed formation and development [18]–[24]. However, identification of all imprinted genes and their biological functions is far from complete, although this can help to understand why parent-of-origin effects are essential for seed development and how they have evolved.

Several genetic models and statistical methods have been derived to estimate the distribution and interactive effects of maternal and zygotic genes through genetic mapping [25]–[28], in which individual quantitative trait loci [QTLs] are mapped with a genetic linkage map constructed by molecular markers. The identification of those QTLs that display parent-of-origin or imprinting effects have received a special attention through linkage analyses in multiple related or unrelated small-sized families [29]–[32] or in large oubred crosses [33]–[35]. The use of an outbred strategy appropriate for plants and animals led to the detection of significant imprinting QTLs for body composition and body weight in pigs [34], [36]–[39] and chickens [40]. However, the inference of imprinting QTLs from an outbred cross may be problematic, because paternally and maternally expressed genetic differences detected can simply be due to different alleles, rather than imprinted effects of the same alleles [41]. Alleles of a given gene, including a marker or QTL, can be different between two outbred parents, because of their heterozygous nature.

To overcome the limitation of the outbred strategy, Cui et al. [42] recently proposed an approach for mapping imprinting QTLs with an F_2_ family, initiated with inbred lines, which allows direct characterization of the maternal and paternal origin of a QTL allele. However, this approach, relying upon the assumption of sex-specific differences in recombination, is limited when such differences do not exist or fail to be estimated. A reciprocal backcross design, as proposed by Clapcott et al. [43], has been shown to be powerful for detecting a major imprinted QTL that controls susceptibility to trypanosomiasis in mice. Cui and colleagues for the first time derived detailed statistical algorithms for testing the existence of imprinting QTLs with such a reciprocal design, making it possible to map imprinting QTLs as a routine endeavor [44], [45]. Based on Cui's [44] idea, we here propose a statistical mapping strategy for integrating the tests of imprinting QTLs into a modeling framework for genetic interactions between the maternal and zygotic (embryo) genomes in seed development. The advantage of this strategy is that, while the inheritance of parent-specific alleles can be traced and therefore the parent-of-origin effects estimated, the interactions between QTLs from the maternal and zygotic genomes can be characterized. The new strategy was used to analyze maize mapping data collected from two pairs of reciprocal backcrosses, derived from two inbred lines displaying sharply contrasting endoreduplication levels. Significant QTLs that control endoreduplication in maize endosperm through genome-genome interactions were detected, and their chromosomal positions and origin-of-parent effects were estimated and tested. Simulation studies were performed to investigate the statistical properties of the new QTL mapping strategy. Finally, we discuss the implications of the new strategy for the characterization of QTLs with maternal-zygotic interactions and parent-of-origin effects in general genetic mapping studies and several areas in which the current strategy can be modified to make it useful.

### Statistical Model

#### Genetic Design

Suppose there are two inbred lines, P_1_ and P_2_, which are crossed to generate an F_1_ progeny population. The F_1_ used as a female and male parent is reciprocally backcrossed to the two original parents, leading to four types of backcrosses, F_1_×P_1_ (labeled as 1), F_1_×P_2_ (labeled as 2), P_1_×F_1_ (labeled as 3), and P_2_×F_1_ (labeled as 4), with sizes n_1_, n_2_, n_3_ and n_4_, respectively. A genetic linkage map can be constructed with molecular markers for each of these four backcrosses. However, we assume that an integrated map is constructed for all four backcrosses. All the offspring from the four backcrosses are measured for a quantitative trait of interest. The linkage map is used to map all possible types of QTLs that control the trait.

#### Quantitative Genetic Model

Consider a segregating QTL with two alleles Q and q for the offspring trait. Thus, at this QTL, the genotypes of the two parental lines, P_1_ and P_2_, and the F_1_ are QQ, qq, and Qq, respectively. In each backcross, this QTL forms two different genotypes, QQ and Qq, or Qq and qq. Table 1 tabulates parental cross types at the QTL, and the offspring segregation types for each backcross. The offspring traits, especially seed traits in plants, are thought to be controlled by two types of QTL, one from the maternal genome and the second from the offspring genome. In this four-backcross design, the maternal QTL genotypes are Qq (backcrosses 1 and 2), QQ (backcross 3) and qq (backcross 4), whereas the offspring (zygotic) QTL genotypes are segregating in each backcross in a way as shown in Table 1. At the end, there are eight different genotype combinations between the maternal and offspring QTL. The eight combinations are sorted into five groups, QQQQ, QQQq, QQqq, Qqqq, and qqqq, according to the relative numbers of alleles Q or q. Each genotypic combination is assigned different additive and dominance effects based on their allelic combinations (Table 2). The additive genetic effect (a) is defined as the effect that is due to the change of the number of an allele Q or q, whereas the dominance effect derives from interactions between different alleles at the QTL. As shown in Table 2, there are three possible dominance effects, d_1_ (the interaction between one Q allele and three q alleles), d_2_ (the interaction between two Q alleles and two q allele), and d_3_ (the interaction between three Q alleles and one q allele).

**Table 1:** Segregation of QTL genotypes in the backcrosses and compositions of genotypic values (*μ_kj_*) for each maternal-offspring QTL genotype in terms of the additive, dominant, maternal by offspring interaction and imprinting effects of the QTL.

Backcross		Parental Genotype		Offspring Genotype	Compositions of (*μ_kj_*)							
No.	Type	Maternal	Paternal		μ	a	d_1_	d_2_	d_3_	δ_1_	δ_2_	λ
1	F_1_×P_1_	Qq	QQ	QQ	1	1	0	0	1	−1	0	0
				q_M_Q_P_	1	0	0	1	0	0	0	−1
2	F_1_×P_2_	Qq	qq	Q_M_q_P_	1	0	0	1	0	0	0	1
				qq	1	−1	1	0	0	0	1	0
3	P_1_×F_1_	QQ	Qq	QQ	1	2	0	0	0	0	0	0
				Q_M_q_P_	1	1	0	0	1	1	0	1
4	P_2_×F_1_	qq	Qq	q_M_Q_P_	1	−1	1	0	0	0	−1	−1
				qq	1	−2	0	0	0	0	0	0

**Table 2:** Genetic compositions of joint maternal-zygotic genotypes at a QTL.

Genotype Combination	Effect	
	Additive	Dominance
QQQQ	2a	0
QQQq	a	d_3_
QQqq	0	d_2_
Qqqq	−a	d_1_
qqqq	−2a	0

In Table 1, it can be seen that the same genotypic combination QQQq may have two types, the first resulting from maternal genotype QQ and zygotic genotype Qq, and the second resulting from maternal genotype Qq and zygotic genotype QQ. Let δ_1_ denote the maternal by zygotic QTL interaction for this genotypic combination, and thus, while the first type of combination is assigned by δ_1_, the second type assigned by −δ_1_. Similarly, we use δ_2_ to denote the maternal by zygotic QTL interaction for genotypic combination Qqqq derived from maternal genotype Qq and zygotic genotype qq, and −δ_2_ to denote such an interaction for genotypic combination Qqqq derived from maternal genotype qq and zygotic genotype Qq. In addition, zygotic genotypes may contribute to the trait differently when the parent-of-origin effect exists. This means that zygotic genotype Q_M_q_F_ performs differently from q_M_Q_F_, where subscripts M and P to specify the maternal and paternal parent from which alleles Q and q arise. Let λ denote the the parent-of-origin effect of the QTL. Thus, it is reasonable to assign the imprinting effects of Q_M_q_F_ and q_M_Q_F_ by λ and −λ, respectively.

Based on the discussions above on the additive, dominance, maternal by zygotic interaction and parent-of-origin effects, we give the genotypic values for each maternal-zygotic QTL genotypic combination which are tabulated in Table 1. By testing each of these genetic effect parameters, we can provide a detailed picture of the genetic control of any offspring trait studied. Below, we will derive a statistical algorithm for estimating and testing these parameters.

#### Likelihood and Algorithm

Interval mapping constructs a mixture model-based likelihood by assuming that the putative QTL is bracketed by two adjacent markers on a linkage group. Let y_1_, y_2_, y_3_ and y_4_ be the phenotypic observations of the trait for backcross, F_1_×P_1_, F_1_×P_2_, P_1_×F_1_ and P_2_×F_1_, respectively. Two possible QTL genotypes in each backcross, one heterozygous and the other homozygous, are symbolized by h and *h̅*, respectively. A likelihood function combining the phenotypic values and marker information of all four backcrosses is constructed as
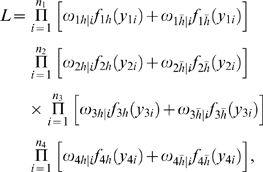
(1)where the proportions of mixture components, *ω_h_*_|*i*_ or *ω*_1*h̅*|*i*_, are expressed as the conditional probability of QTL genotype h or *h̅* given the marker genotype of individual *i* in a backcross, and the mixture components are modelled by a normal distribution density function, *f_kj_*(*y_ki_*), with means expressed as genotypic values (*μ_kj_*) in Table 1 and variances σ_1_^2^, σ_2_^2^, σ_3_^2^, σ_4_^2^ for each backcross, respectively. The conditional probabilities are derived in terms of the ratio (θ) of the recombination fraction between the left marker and QTL over that between the two markers by assuming that there is no double crossover [46]. Thus, by estimating θ, the position of the QTL can be determined. The standard EM algorithm [47] can be used to obtain the maximum likelihood estimates (MLE) of the unknown parameters 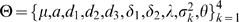 contained in the likelihood (see the **Methods**).

### Hypothesis Testing

Following parameter estimation, several hypotheses should be tested. The hypothesis about the presence of a QTL segregating in the backcrosses is formulated as
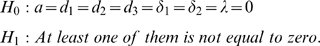
(2)The difference between the log-likelihood functions under the null and alternative hypotheses are calculated. But the distribution of this log-likelihood ratio (LR) is not known because of the violation of regularity conditions for the mixture model (1). For this reason, a commonly used empirical approach based on permutation tests by reshuffling the relationships between the marker genotypes and phenotypes [48] is used to determine the critical threshold, in order to judge whether there is a QTL for the offspring trait.

If a QTL is found to be present, then we need to test whether its additive, dominance, maternal by offspring interaction and imprinting effects are significant by formulating the following tests:
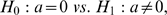
(3)

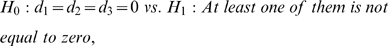
(4)

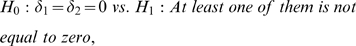
(5)

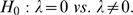
(6)


If the null hypothesis of (5) is rejected, this means that the detected QTL may display a significant effect due to maternal-zygotic interactions. Similarly, if the null hypothesis of (6) is rejected, this indicates that the QTL detected may be imprinted and it therefore can be called an imprinting QTL. The sign of λ reflects the direction of the imprinting effect of this QTL. If λ is positive, this means that the maternally-derived allele is expressed and thus the paternally-derived allele is imprinted. The inverse is true for a negative λ value. If the maternally- or paternally-derived allele is completely imprinted, we should have
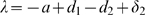
(7)

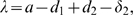
(8)respectively. Thus, using these two equalities as a null hypothesis can test whether the imprinting QTL is completely imprinted. The rejection of these null hypotheses implies that the QTL is partially maternally or paternally imprinted.

For all the hypotheses (3)–(8), the log-likelihood ratios calculated under the null and alternative hypotheses are thought to asymptotically follow a χ^2^-distribution with the degree of freedom equal to the difference in the number of unknown parameters between the null and alternative hypotheses.

It is important to estimate the relative proportion of the total phenotypic variance explained by the total genetic effect of a QTL. The genotypic variance among the two maternal-zygotic combinations within each backcross is calculated by 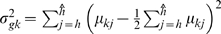. The proportions of the total phenotypic variance contributed by the QTL for all the four backcrosses are calculated as
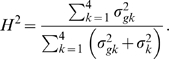
(9)


## Results

**Figure 1:**
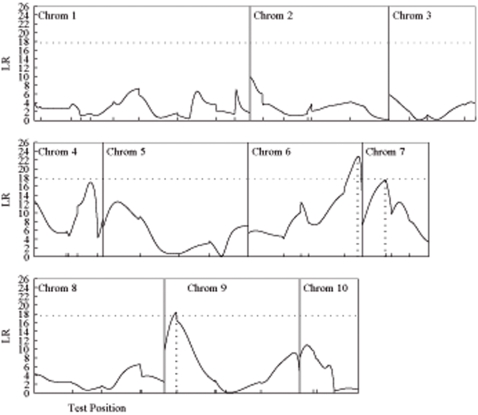
The profile of log-likelihood ratios (LR) between the full (there is a QTL) and reduced (there is no QTL) model for mean ploidy across an integrated linkage map constructed by the four backcrosses in maize (Coelho et al. 2007). The peaks of the profile correspond to the MLEs of the QTL positions indicated by the vertical dot lines. The genome-wide threshold value (17.67) for claiming the existence of QTL determined from 1000 permutation tests is given as the horizonal dot line. The positions of markers on the chromosomes are shown by the ticks.

**Figure 2:**
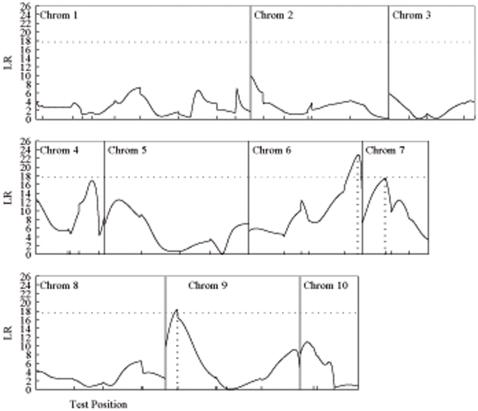
The profile of log-likelihood ratios (LR) between the full (there is a QTL) and reduced (there is no QTL) model for the percentage of endoreduplicated nuclei across an integrated linkage map constructed by the four backcrosses in maize (Coelho et al. 2007). The peaks of the profile correspond to the MLEs of the QTL positions indicated by the vertical dot lines. The genome-wide threshold value (17.87) for claiming the existence of QTL determined from 1000 permutation tests is given as the horizonal dot line. The positions of markers on the chromosomes are shown by the ticks.

Mean ploidy and the percentage of endoreduplicated nuclei, measured for two pairs of reciprocal backcrosses, initiated with two inbred lines, Sg18 and Mo17, are two physiological parameters that describe the level of endoreduplication in the embryo. They displayed higher values for the Sg18 (15.2C and 72.3%) than the Mo17 (9.8C and 54.8%) parent. Tremendous variation was observed in the degree of endoreduplication for each backcross [49]. Our mapping strategy was used to genome-wide map and identifies QTLs that trigger maternal-zygotic interactions and parent-of-origin effects on endoreduplication traits in the endosperm. Figures 1 and 2 illustrate the profiles of the LR for testing the presence of such QTL based on hypothesis (2). The peaks of the LR curve beyond the genome-wide critical threshold shown in the figures correspond to the locations of significant QTLs. Table 3 tabulates the estimated chromosomal positions of each QTL detected and its additive, dominance, maternal-zygotic interaction and imprinting effects on endoreduplication.

**Table 3:** The MLEs of the QTL positions and additive (a), dominance (d_1_, d_2_, and d_3_), maternal-zygotic interaction (δ_1_ and δ_2_) and imprinting effects (λ) on mean ploidy and the percentage of endoreduplicated nuclei via a joint analysis of the four backcrosses derived from the Sg18 and Mo17 inbred lines.

Chromosome	Marker Interval	Parameter Estimation								
		μ	a	d_1_	d_2_	d_3_	δ_1_	δ_2_	λ	LR
**Mean ploidy**										
6	umc2324-umc2059	10.92	1.10	−0.78	2.75	1.85	1.70	1.67	−2.46	22.76
7	umc1066-dupssr9	11.60	1.06	−0.70	−0.21	0.34	−0.10	0.84	−0.31	17.51
9	umc1430-umc1040	10.74	0.92	0.69	0.08	1.35	0.34	0.29	0.33	18.36
**Percentage of endoreduplicated nuclei**										
7	dupssr9	62.21	5.08	1.83	−2.32	−3.34	1.94	4.40	3.59	17.48

LR is the log-likelihood ration that tests for the existence of a significant QTL.

Three QTLs responsible for mean ploidy were detected between umc2324 and umc2059 on chromosome 6, umc1066 and dupssr9 on chromosome 7 and umc1430 and umc1040 on chromosome 9 (Fig. 1; Table 3). All these QTLs display a significant additive genetic effect (Table 4), at each of which parent Sg18 contributes a favorable allele to increased mean ploidy values (Table 3). Overall, compared to the additive effects, the dominance genetic effects are significant to a lesser extent. The QTLs on chromosomes 6 and 7 exhibit significant maternal-zygotic interaction effects, whereas the QTL on chromosome 9 does not. A significant imprinting effect was observed for the QTL on chromosome 6, but not for those on chromosomes 7 and 9 (Table 3). The negative estimate of the imprinting effects for the chromosome 6 QTL suggests (Table 3) that the paternally-derived allele at this QTL is expressed while the maternally-derived allele is imprinted.

**Table 4:** Log-likelihood ratio values for hypothesis tests regarding the additive (a), dominance (d_1_, d_2_, and d_3_), maternal-zygotic interaction (δ_1_ and δ_2_) and imprinting effects (λ) of the detected QTLs on mean ploidy and percentage of endoreduplicated nuclei.

Hypothesis Testing		Mean Ploidy			% End. Nuclei
Parameter	H_0_	Chr. 6	Chr.7	Chr. 9	Chr. 7
Additive	a = 0	90.62^***^	95.77^***^	78.17^***^	140.38^***^
Dominance	d_1_ = d_2_ = d_3_ = 0	19.65^**^	9.77^*^	25.65^***^	16.88^***^
Maternal-Zygotic	δ_1_ = δ_2_ = 0	16.69^***^	19.73^***^	0.03^ns^	36.94^***^
Imprinting	λ = 0	12.00^***^	1.96^ns^	0.00^ns^	23.30^***^

Note: ^*^Significant at p<0.05, ^**^Significant at p<0.01, ^***^Significant at p<0.001, ^ns^Nonsignificant.

One QTL was detected for the percentage of endoreduplicated nuclei near dupssr9 on chromosome 7 (Fig 2; Table 3). The additive effect at this QTL is significant, with the favorable allele contributed by parent Sg18 to an increased percentage of endoreduplicated nuclei. This QTL displays significant additive, dominance, maternal-zygotic interaction and parent-of-origin effects due to the imprinting of the paternally-derived allele (Table 4). It is interesting to note that the QTL on chromosome 7 for the percentage of endoreduplicated nuclei is located at a similar position on the same chromosome as one affecting mean ploidy, suggesting that this is a pleiotropic QTL with an effect on the two different but correlated endoreduplication traits. This pleiotropic QTL triggers significant additive and maternal-zygotic interaction effects on both endoreduplication traits, but only displays significant dominance and imprinting effects on the percentage of endoreduplicated nuclei (Table 4).

To examine the statistical behavior of the new mapping strategy based on two pairs of reciprocal backcrosses, F_1_×P_1_, F_1_×P_2_, P_1_×F_1_ and P_2_×F_1_, we performed simulation studies which include several representative schemes with different imprinting degrees (none, small, mediate and large), different maternal-zygotic interaction effects (none and large), different heritabilities (0.1 and 0.4), and different sample sizes (100 and 400 for each backcross). We simulated a linkage group of length 200 cM constructed by 11 equally-spaced markers. Suppose there is a QTL located at 36 cM from the first marker. The genetic parameters of this QTL used to simulate the phenotypic data under different schemes are given in Tables 5 and 6, with the residual errors assumed to be normally distributed with mean zero and variance adjusted for a given heritability level. Each simulation scheme is repeated 1000 times to estimate the means and standard deviations of the parameter estimates that include the QTL location, genetic effects and residual variances.

**Table 5:** MLEs of the position and effect parameters for a QTL with varying imprinting effects (λ) under simulation schemes of different heritabilities (H^2^) and sample sizes for each backcross (n).

Scheme	Position	μ	a	d_1_	d_2_	d_3_	δ_1_	δ_2_	λ	σ_1_^2^	σ_2_^2^	σ_3_^2^	σ_4_^2^	Power 1	Power 2	Type I Error Rate
True value	36	10	1	0.6	0.4	0.3	1.5	1.8								
λ = 0																
1	36.96 (6.76)	10.01 (0.09)	1.00 (0.05)	0.60 (0.15)	0.38 (0.16)	0.30 (0.21)	1.51 (0.14)	1.81 (0.15)	−0.01 (0.14)	0.79 (0.11)	2.20 (0.32)	1.45 (0.24)	0.09 (0.01)	-	100	4
2	35.56 (1.61)	10.00 (0.04)	1.00 (0.02)	0.60 (0.08)	0.40 (0.08)	0.31 (0.09)	1.50 (0.06)	1.80 (0.07)	0.00 (0.06)	0.81 (0.06)	2.25 (0.14)	1.43 (0.11)	0.09 (0.01)	-	100	7
3	35.68 (1.40)	10.00 (0.03)	1.00 (0.02)	0.60 (0.06)	0.40 (0.06)	0.30 (0.08)	1.50 (0.05)	1.80 (0.06)	0.00 (0.05)	0.13 (0.02)	0.37 (0.06)	0.24 (0.03)	0.01 (0.00)	-	100	5
4	35.80 (0.07)	10.00 (0.02)	1.00 (0.01)	0.60 (0.03)	0.40 (0.03)0(0.04)	0.29 (0.04)	1.50 (0.03)	1.80 (0.03)	0.00 (0.03)	0.14 (0.01)	0.37 (0.03)	0.24 (0.02)	0.01 (0.00)	-	100	3
λ = 0.3																
1	36.96 (6.76)	10.02 (0.14)	1.01 (0.07)	0.60 (0.15)	0.38 (0.16)	0.29 (0.24)	1.51 (0.14)	1.81 (0.11)	0.30 (0.09)	0.20 (0.03)	1.08 (0.15)	2.74 (0.45)	0.55 (0.08)	96	100	-
2	35.56 (1.61)	10.00 (0.06)	1.00 (0.04)	0.60 (0.07)	0.40 (0.08)	0.31 (0.11)	1.51 (0.06)	1.80 (0.05)	0.30 (0.04)	0.20 (0.01)	1.10 (0.07)	2.71 (0.20)	0.56 (0.04)	100	100	-
3	35.66 (1.72)	10.00 (0.05)	1.00 (0.03)	0.60 (0.06)	0.40 (0.06)	0.29 (0.08)	1.49 (0.05)	1.80 (0.04)	0.31 (0.04)	0.03 (0.00)	0.18 (0.03)	0.44 (0.06)	0.09 (0.01)	100	100	-
4	35.8 (0.07)	10.00 (0.03)	1.00 (0.01)	0.60 (0.03)	0.40 (0.03)	0.29 (0.05)	1.50 (0.03)	1.80 (0.02)	0.30 (0.02)	0.03 (0.00)	0.18 (0.01)	0.45 (0.04)	0.09 (0.01)	100	100	-
λ = −0.6																
1	35.72 (4.97)	9.99 (0.05)	1.00 (0.03)	0.61 (0.27)	0.42 (0.22)	0.31 (0.18)	1.51 (0.17)	1.82 (0.23)	−0.61 (0.23)	3.20 (0.47)	5.46 (0.78)	0.09 (0.01)	0.36 (0.06)	79	100	-
2	36.12 (1.86)	10.00 (0.03)	1.00 (0.01)	0.63 (0.11)	0.40 (0.10)	0.29 (0.09)	1.50 (0.09)	1.81 (0.10)	−0.58 (0.10)	3.21 (0.24)	5.73 (0.39)	0.09 (0.01)	0.36 (0.03)	99	100	-
3	35.80 (1.36)	10.00 (0.02)	1.00 (0.01)	0.58 (0.09)	0.40 (0.09)	0.30 (0.08)	1.50 (0.07)	1.79 (0.09)	−0.6 (0.08)	0.54 (0.08)	0.95 (0.15)	0.02 (0.00)	0.06 (0.01)	100	100	-
4	35.82 (0.72)	10.00 (0.01)	1.00 (0.01)	0.60 (0.04)	0.41 (0.05)	0.30 (0.04)	1.50 (0.04)	1.80 (0.04)	−0.59 (0.05)	0.54 (0.04)	0.96 (0.07)	0.02 (0.00)	0.06 (0.00)	100	100	-
λ = 0.8																
1	36.10 (4.08)	9.97 (0.22)	1.00 (0.11)	0.62 (0.23)	0.43 (0.23)	0.34 (0.34)	1.51 (0.18)	1.81 (0.11)	0.80 (0.03)	0.09 (0.01)	0.09 (0.01)	5.62 (0.76)	2.22 (0.35)	100	100	-
2	35.86 (1.81)	10.00 (0.10)	1.00 (0.05)	0.61 (0.11)	0.40 (0.11)	0.31 (0.15)	1.51 (0.09)	1.80 (0.05)	0.80 (0.01)	0.09 (0.01)	0.09 (0.01)	5.68 (0.42)	2.25 (0.17)	100	100	-
3	36.04 (1.56)	10.01 (0.08)	1.01 (0.04)	0.60 (0.09)	0.39 (0.08)	0.28 (0.13)	1.50 (0.07)	1.80 (0.05)	0.80 (0.01)	0.01 (0.00)	0.01 (0.00)	0.97 (0.16)	0.37 (0.05)	100	100	-
4	35.74 (0.88)	10.00 (0.04)	1.00 (0.02))	0.60 (0.04)	0.40 (0.04)	0.30 (0.06)	1.50 (0.03)	1.80 (0.02)	0.80 (0.01)	0.02 (0.00)	0.02 (0.00)	0.96 (0.07)	0.38 (0.03)	100	100	-

The numbers in the parentheses are the square roots of the mean square errors of the MLEs calculated from 100 simulation replicates. The power to detect the imprinting effect (1) and maternal-zygotic interaction effect (2) of a QTL, and the Type I error rate under no imprinting effect were also given.

Note: Simulation schemes: (1) H^2^ = 0.1 and n = 100, (2) H^2^ = 0.1 and n = 400, (3) H^2^ = 0.4 and n = 100, and (4) H^2^ = 0.4 and n = 400, where n denotes the sample size of each of the four backcrosses. For λ = 0, the residual variances σ_1_^2^, σ_2_^2^, σ_3_^2^ and σ_4_^2^ are 0.81, 2.25, 1.44, 0.09 for H^2^ = 0.1, and 0.14, 0.38, 0.24, 0.02 for H^2^ = 0.4, respectively. These values are 0.20, 1.10, 2.72, 0.56 for H^2^ = 0.1, and 0.03, 0.18, 0.45, 0.09 for H^2^ = 0.4 for λ = 0.3; 3.24, 5.76, 0.09, 0.36 for H^2^ = 0.1, and 0.54, 0.96, 0.02, 0.06 for H^2^ = 0.4 for λ = −0.6; and 0.09, 0.09, 5.76, 2.25 for H^2^ = 0.1, and 0.02, 0.02, 0.96, 0.38 for H^2^ = 0.4 for λ = 0.8.

**Table 6:** MLEs of the position and effect parameters for a QTL without maternal-zygotic interaction effect under simulation schemes of different heritabilities (H^2^) and sample sizes for each backcross (n).

Scheme	Position	μ	a	d_1_	d_2_	d_3_	δ_1_	δ_2_	λ	σ_1_^2^	σ_2_^2^	σ_3_^2^	σ_4_^2^	Power	Type I Error Rate
True value	36	10	1	0.6	0.4	0.3	0	0	−0.6						
1	35.72 (5.96)	10.04 (0.27)	0.99 (0.14)	0.57 (0.46)	0.37 (0.27)	0.29 (0.28)	0.01 (0.15)	−0.01 (0.25)	−0.60 (0.04)	0.20 (0.03)	0.09 (0.01)	3.84 (0.60)	10.73 (1.46)	100	9
2	36.18 (1.98)	10.00 (0.14)	1.00 (0.08)	0.59 (0.24)	0.40 (0.14)	0.32 (0.14)	0.01 (0.07)	0.01 (0.12)	−0.60 (0.02)	0.20 (0.01)	0.09 (0.01)	3.78 (0.28)	10.91 (0.78)	100	5
3	35.44 (1.75)	10.01 (0.10)	0.99 (0.06)	0.57 (0.17)	0.39 (0.11)	0.29 (0.11)	−0.01 (0.06)	0.01 (0.10)	−0.60 (0.02)	0.03 (0.00)	0.01 (0.00)	0.62 (0.09)	1.78 (0.28)	100	3
4	35.72 (0.80)	10.01 (0.06)	0.99 (0.03)	0.59 (0.09)	0.39 (0.06)	0.30 (0.06)	0.00 (0.03)	−0.00 (0.05)	−0.60 (0.01)	0.03 (0.00)	0.02 (0.00)	0.63 (0.04)	1.82 (0.14)	100	5

The numbers in the parentheses are the square roots of the mean square errors of the MLEs calculated from 100 simulation replicates. The power to detect the imprinting effect of a QTL and the Type I error rate for the detection of maternal-zygotic interaction effect were also given. Note: Simulation schemes: (1) H^2^ = 0.1 and n = 100, (2) H^2^ = 0.1 and n = 400, (3) H^2^ = 0.4 and n = 100, and (4) H^2^ = 0.4 and n = 400, where n denotes the sample size of each of the four backcrosses. The residual variance σ_1_^2^, σ_2_^2^, σ_3_^2^ and σ_4_^2^ are 0.20, 0.09, 3.80, 10.89 for H^2^ = 0.1, and 0.03, 0.02, 0.63, 1.82 for H^2^ = 0.4, respectively.

Results from simulation studies suggest that a modest sample size (100) and heritability level (0.1) can be adequate to provide accurate and precise estimates of the position of a QTL, its additive genetic effect and two maternal-zygotic interaction effects, and nuisance parameters-the overall mean and residual variances (Table 4). To obtain comparable estimation precision for three dominance effects and the imprinting effect, a large sample size (say 400) and/or a large heritability (say 0.4) is needed (Table 4). Estimation accuracy and precision of all the parameters can be dramatically improved with increasing sample sizes and heritabilities. In general, there is adequate power to detect the imprinting effect of a QTL for a modest sample size and heritability level, even when the imprinting effect is small (Table 4). For data that contain no imprinting effect, our mapping strategy may generate some type I errors (3–7%). In other words, there may be a small probability to infer the imprinting effects of a QTL by the new model, even if there is actually no such effect.

The mapping strategy was also examined in terms of the power for detecting two maternal-zygotic interaction effects of a QTL. A modest sample size (100) and heritability (0.1) can assure adequate power for the detection of such interaction effects (Table 4). Also, such a sample size and/or heritability level can well avoid a large type I error (<10%) for detecting maternal-zygotic interaction effects (Table 5). The estimation precision of the main genetic effects, especially three dominance effects, is affected by the size of imprinting effect; a large imprinting effect is associated with poorer estimation precision (Table 4). The imprinting effect does not affect the estimation precision of two maternal-zygotic interaction effects because the latter was found to be similar when different imprinting effects were assumed (Table 4). Yet, the sizes of maternal-zygotic interaction effects affect the estimation precision of the imprinting effect; larger interactions effects lead to poorer estimation of the imprinting effect (Tables 4 *vs.* 5).

## Discussion

Proper development of a seed in flowering plants requires the coordination among its embryo, endosperm and maternal components [1]. For this reason, an understanding of how these three components interact in a coordinated way to regulate seed development has been a long-standing topic of a great interest to plant geneticists and developmental biologists [2]–[6]. Wu and group are among the first to develop statistical models and algorithms for characterizing maternal-zygotic effects of quantitative trait loci (QTLs) on seed development [25], [27], [28], [50], and further used these models to map genome-genome interactive QTLs that control endosperm traits in rice [26] and maize [49].

In this article, we propose a new statistical strategy for integrating the concept of maternal-zygotic interactions into a mapping framework for the detection of imprinting QTLs. Genetic imprinting has been previously thought to occur rarely, but studies have increasingly demonstrated an important role of this phenomenon in regulating and directing trait expression and development. de Koning et al. [34] detected four imprinting QTLs involved in body composition in pigs that are located in the region of the *Sus scrofa* candidate genes. In flowering plants, the proper development of the embryo requires the coordinated expression of a different nutritive tissue, the triploid endosperm [2]. Imprinted genes have been found to affect the development and size of the endosperm [16], [18]. In *Arabidopsis*, Vielle-Calzada and colleagues reported that most of the paternal genome is silenced during the early seed development, suggesting that the embryo and endosperm are mainly under maternal control at early stages of development [17], [24]. These studies underscore the value of developing a statistical model that empowers researchers to identify the distribution and effects imprinted genes.

Our mapping strategy was founded on Cui's [43] model design in which reciprocal backcrosses derived from inbred lines are jointly modelled by a maximum likelihood approach. This design of reciprocal backcrosses, first conceived by Clapcott et al. [43], uses the F_1_ individual as both maternal and paternal parents in backcrossing. Since inbred lines have contributed enormously to the genetic mapping of quantitative traits, the strategy proposed here can serve as a routine tool for genetic mapping of QTLs responsible for seed development. This strategy was used to analyze endoreduplication traits in two pairs of reciprocal backcrosses of maize inbreds, leading to the identification of a few imprinted QTLs that trigger maternal parent-of-origin effects on two measures of endosperm endoreduplication - mean ploidy and the percentage of endoreduplicated nuclei. Chromosomes 6, 7 and 9 were found to harbor QTLs for mean ploidy, whereas the QTL on chromosome 7 was also observed to affect the percentage of endoreduplicated nuclei. There seems to be a strong signal for the existence of a pleiotropic QTL between markers umc1066 and dupssr9 on chromosome 7 that jointly controls two endoreduplication traits, although its control mechanisms are trait-dependent. For example, this QTL triggers significant additive, dominance, maternal-zygotic interaction and imprinting effects on the percentage of endoreduplicated nuclei, but it affects mean ploidy only through the additive and maternal-zygotic interaction effects (Table 4). Many of these results about QTL detection are consistent with those obtained from triploid and QTL epistatic models [49].

Statistical precision of the results obtained by the new mapping strategy was analyzed by simulation studies. Under a modest sample size and heritability, the strategy was found to be able to provide a reasonable estimation of the additive genetic and maternal-zygotic interaction effects of a QTL, but, in order to precisely estimate the dominance and imprinting effects, increased sample sizes and/or heritabilities are needed. Detailed simulation studies were performed to investigate the statistical power of the new strategy for the detection of imprinting and maternal-zygotic interaction effects. In practice, some caution is needed to avoid a false positive rate for detecting imprinting and maternal-zygotic interaction effects by increasing sample sizes and/or heritabilities.

The second caution about the inference of imprinting effects is that the QTLs detected to interact between the maternal and zygotic genomes may be due to the maternal, paternal or zygotic effects of the QTLs because these effects also contribute to variation in endosperm traits. Thus, to make the best statistical inference, the maternal-zygotic interaction model presented in this article should be used in conjunction with the models for characterizing the maternal, paternal, embryo, and endosperm effects. Table 7 listed the genotypic compositions of the maternal and paternal models in which different parameter formulations allow the two models distinguishable. The genotypic compositions of the embryo and endosperm models are shown in Table 8, where these two models are not identifiable although different types of parameters are estimated. Thus, by calculating model selection criteria, such as AIC or BIC, for the interaction (Table 1), maternal (Table 7), paternal (Table 7), and embryo or endosperm models (Table 8), the optimal one that best fits the data can be chosen. To make the embryo and endosperm models distinguishable, a more informative genetic design, like a two-stage hierarchical genotyping design [51], is needed.

**Table 7:** The maternal and paternal models for the genetic control of endosperm traits in the backcrosses.

Backcross		Maternal Model		Paternal Model	
No.	Type	Genotype	Value	Genotype	Value
1	F_1_×P_1_	Qq	μ_M_+d_M_	Qq	μ_P_+a_P_
2	F_1_×P_2_	Qq	μ_M_+d_M_	Qq	μ_P_−a_P_
3	P_1_×F_1_	QQ	μ_M_+a_M_	Qq	μ_P_+d_P_
4	P_2_×F_1_	qq	μ_M_−a_M_	Qq	μ_P_+d_P_

The two models include the overall mean (μ_M_ and μ_P_), additive (a_M_ and a_P_) and dominance effects (d_M_ and d_P_ ), respectively.

**Table 8:** The embryo and endosperm models for the genetic control of endosperm traits in the backcross.

Backcross		Paternal Genotype		Embryo Model		Endosperm Model	
No.	Type	Maternal	Paternal	Genotype	Value	Genotype	Value
1	F_1_×P_1_	Qq	QQ	QQ	μ_m_+a_m_	QQQ	μ_n_+3/2a_n_
				q_M_Q_P_	μ_m_+d_m_−i_m_	q_M_q_M_Q_P_	μ_n_−a_n_+d_n1_
2	F_1_×P_2_	Qq	qq	Q_M_q_P_	μ_m_+d_m_+i_m_	Q_M_Q_M_q_P_	μ_n_+a_n_+d_n2_
				qq	μ_m_−a_m_	qqq	μ_n_−3/2a_n_
3	P_1_×F_1_	QQ	Qq	QQ	μ_m_+a_m_	QQQ	μ_n_+3/2a_n_
				Q_M_q_P_	μ_m_+d_m_+i_m_	Q_M_Q_M_q_P_	μ_n_+a_n_+d_n2_
4	P_2_×F_1_	qq	Qq	q_M_Q_P_	μ_m_+d_m_−i_m_	q_M_q_M_Q_P_	μ_n_−a_n_+d_n1_
				qq	μ_m_−a_m_	qqq	μ_n_−3/2a_n_

The embryo model includes the overall mean (μ_m_), additive (a_m_), dominance (d_m_), and imprinting effects (i_m_), whereas the endosperm model includes the overall mean (μ_n_), additive (a_n_), q_M_q_M_ over Q_P_ dominance (d_n1_), and Q_M_Q_M_ over q_P_ dominance effects (d_n2_).

Although the mechanisms for genetic imprinting are not totally understood, this phenomenon is thought to offer an evolutionary advantage through the maintenance of greater genetic variation, as opposed to non-imprinting of a gene. An imprinting QTL can function in a coordinated network of gene-gene and gene-environment interactions. For some critically important genes that regulate seed growth, there is a critical window during fertilized zygote development in which environmental exposure alters genomic imprinting. These epigenetic changes can have significant phenotypic consequences, including increased or reduced seed size in flowering plants [20], [23]. In addition, our model was constructed on the same QTL that is segregating in the maternal and zygotic genomes. Although the results from a limited number of genetic studies suggested that it is possible for the same maternal and zygotic QTL to regulate the biological function of embryo or endosperm development, the model can be extended, with no technical difficulty, to model the epistatic interactions between different QTLs from the maternal and zygotic genomes [25], [27], [28]. It is worthwhile developing new statistical models for the detection of interaction regulatory genes that affect the imprinting expression of any QTLs involved in a genetic network composed of the maternal and zygotic genomes.

## Materials and Methods

### Plant Materials

An F_1_ progeny was produced by crossing the popcorn Sg18 inbred line having a high level of endoreduplication with the Midwestern Mo17 dent inbred line having a lower level of endoreduplication. The F_1_ individuals, as a maternal and paternal parent, were backcrossed to Sg18 and Mo17 to generate two pairs of reciprocal backcrosses, F_1_×Sg18 (labeled as 1), F_1_×Mo17 (labeled as 2), Sg18×F_1_ (labeled as 3), and Mo17×F_1_ (labeled as 4), with 89, 82, 92, and 85 individuals, respectively [48]. Developing kernels from each backcross were harvested from the middle of well-filled ears at 16 days after pollination (DAP). Endosperms were dissected and analyzed by flow cytometry, and their corresponding embryos were rescued by tissue culture and grown to seedlings, as described by Dilkes et al. [52].

Seedlings of the backcrosses progeny were lyophilized with a speed vacuum dryer at −40°C. DNA was prepared by the hexadecyltrimethyl-ammonium bromide method and diluted to a final concentration of approximately 10 ng/ul for PCR reactions. Simple sequence repeat (SSR) primers were purchased from Research Genetics (Huntsville, AL) or Invitrogen (Carlsbad, CA). The primer sequences are available in the Maize Genomic Database (http://www.maizegdb.org/ssr.php). Of approximately 500 SSR primer pairs screened, only 65 amplified clear and unambiguous polymorphic DNA fragments in the backcrosses initiated with Sg18 and Mo17. An integrated linkage group composed of the 10 maize chromosomes was constructed for the four backcrosses with the 65 SSR markers. Although the average interval between markers for the linkage map was close to 16 cM, there were some gaps for a few chromosomes in which no linked markers were detected.

Two parameters were used to estimate the degree of endoreduplication in endosperm of the backcross progeny and parental inbred lines, mean ploidy and percentage of endoreduplicated nuclei [52]. DNA content was calculated as mean ploidy by multiplying the nuclear ploidy level by the number of nuclei in each ploidy class. The percentage of endoreduplicated nuclei was calculated as the number of nuclei with 6C and greater DNA content, divided by the total number of nuclei, and multiplied by 100. With the marker and phenotypic data collected for the reciprocal backcross design, our imprinting model allows for detecting and testing iQTL located in the embryo genome that affect endosperm traits.\\

### Statistical Algorithm

A detailed EM algorithm is described to obtain the MLEs of the backcross-specific overall mean, the QTL position, QTL effects and residual variances. Taking the log of the likelihood (1) for the unknown parameters Θ leads to
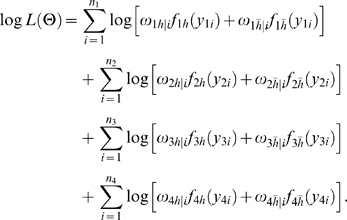
Define the posterior probability of individual *i* to carry a heterozygous QTL genotype *h* in backcross *k* (*k* = 1, 2, 3, 4) as
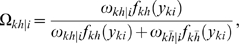
(10)where *h̅* denotes the homozygous QTL genotype.

We derive the log-likelihood equations for each unknown in Θ in terms of the posterior probabilities. These are written as
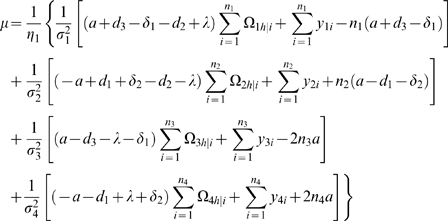


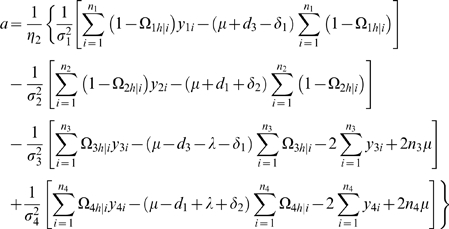


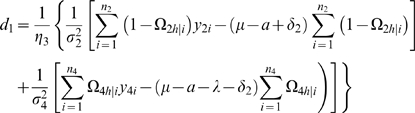


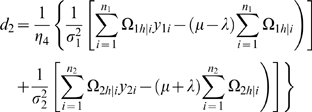


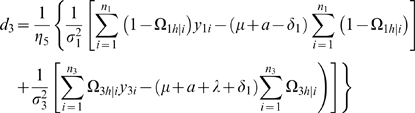


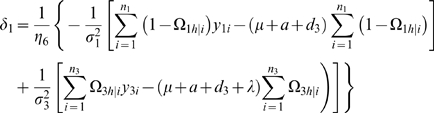


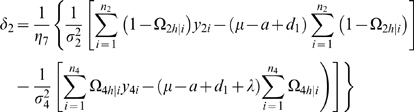


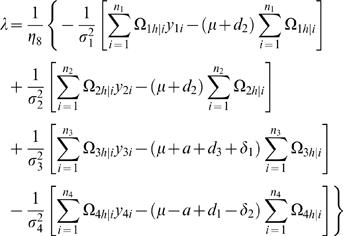


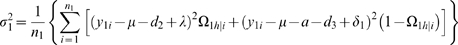


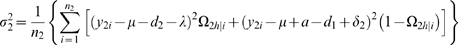


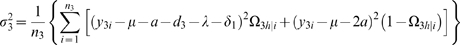


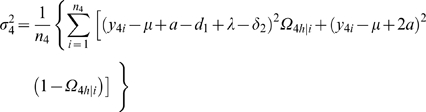
(11)

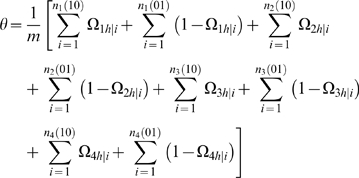
(12)where
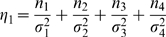


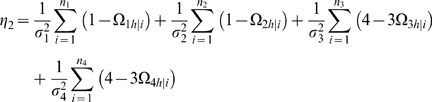


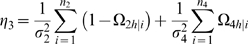


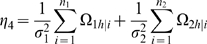


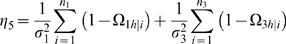


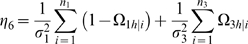


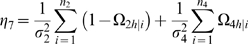


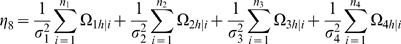
and
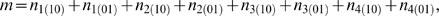
the numbers in the parentheses denote the sample sizes of the recombinant genotype of the two interval markers that bracket the QTL.

The computational procedure is set up as follows. In the E step, calculate the posterior probabilities using equation (10). In the M step, estimate the unknown parameters using the log-likelihood equations (11)–(12). These two steps are iterated until the estimates of the parameters are stable. In practical computation, the QTL position parameter (θ) can be viewed as a fixed parameter because a putative QTL can be searched at every 1 or 2 cM on a map interval bracketed by two markers throughout the entire linkage map. The log-likelihood ratio test statistic for a QTL at a particular map position is displayed graphically to generate a likelihood map or profile. The genomic position that corresponds to a peak of the profile is the MLE of the QTL location.
